# The Cardiovascular Profile Score in Patients with Non-immune Hydrops Fetalis and Cardiac Anomalies — a Pilot Study

**DOI:** 10.1007/s43032-023-01216-w

**Published:** 2023-03-29

**Authors:** Anna Dionysopoulou, Etienne Pirih, Doris Macchiella, Anja Fruth, Antje Jahn-Eimermacher, Christoff Kampmann, Eva Mildenberger, Catharina Whybra

**Affiliations:** 1 Department of Obstetrics and Gynecology, University Medical Center of the Johannes Gutenberg University Mainz, Langenbeckstrasse 1, 55131 Mainz, Germany; 2 Department of Neonatology, University Medical Center of the Johannes Gutenberg University Mainz, Mainz, Germany; 3 Department of Mathematics and Natural Sciences, University of Applied Sciences, Darmstadt, Germany; 4 Department of Pediatric Cardiology, University Medical Center of the Johannes Gutenberg University Mainz, Mainz, Germany

**Keywords:** Non-immune hydrops fetalis, Cardiac anomalies, Cardiovascular profile score, Fetal outcome

## Abstract

The purpose of this paper is to explore whether the cardiovascular profile score (CVPS) correlates with fetal outcome in patients with non-immune hydrops fetalis (NIHF) and cardiac anomalies. In this retrospective study, we included fetuses with NIHF and the suspicion of a cardiac anomaly in prenatal ultrasound. The CVPS was calculated using information obtained by fetal echocardiographic examination. Feto-neonatal mortality (FNM) was defined as intrauterine fetal demise or death in the first 6 months of life. We reviewed 98 patients, who were referred to the Department of Obstetrics and Gynecology of the Johannes Gutenberg University in Mainz with the diagnosis of NIHF between January 2007 and March 2021. By eighteen of them, the suspicion of a cardiac anomaly was raised. After exclusion of six pregnancies (one termination of pregnancy and five because of incomplete data), 12 cases were left for analysis. Mean gestational age at which the CVPS was calculated was 29 + 2 weeks. Two fetuses died in utero. Of the remaining ten hydropic fetuses, three newborns died in the neonatal period, and seven survived after a 6-month surveillance period. Median CVPS of all fetuses was 6 points. Surviving fetuses showed statistically significantly higher CVPS values (median 8 points) than fetuses with FNM (median 5 points, *p* value = 0.009). Our results point towards a positive association between CVPS and fetal outcome in fetuses with NIHF and cardiac anomalies. The CVPS appears to be a useful marker in the assessment of heart failure in utero.

**Graphical Abstract:**

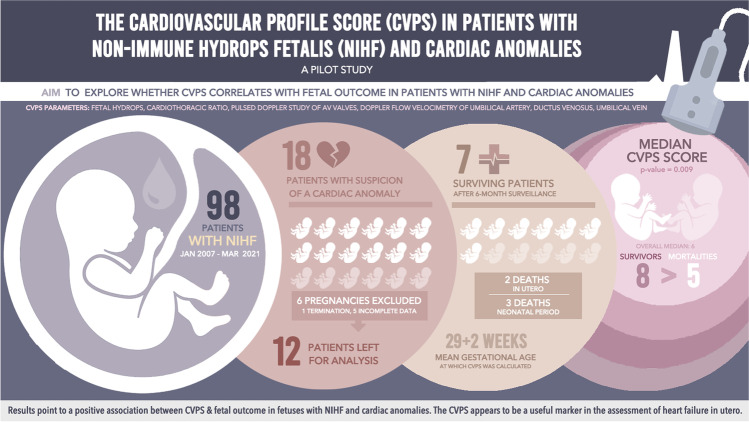

## Introduction

The term hydrops fetalis describes a serious fetal condition, in which abnormal accumulation of fluid in two or more fetal body cavities (thorax, abdomen, pericardium, or skin edema) occurs. Its incidence ranges in the literature between 1:1500 and 1:3000 pregnancies [1, 2]. In terms of classification, the presence or absence of maternal antibodies causing hemolytic disease of the fetus, is used in differentiating between immune and non-immune cases. The implementation of antenatal prophylaxis with anti-d immunoglobulin to Rhesus negative pregnant women has led to a considerable reduction in the incidence of immune hydrops fetalis. As a result, 85–90% of cases of hydrops fetalis are nowadays attributed to non-immune causes [3, 4].

Non-immune hydrops fetalis (NIHF) is not considered a disease itself, but rather a clinical state and the end-stage of a variety of disorders. After the diagnosis of NIHF is made, the next step is to establish the etiology. The Society for maternal–fetal medicine (SMFM), outlines that a detailed anatomic examination of the fetus, including a fetal echocardiographic examination, a Doppler interrogation of the middle cerebral artery, as well as diagnostic invasive testing for fetal karyotype or chromosomal microarray analysis, should be performed, irrespective of the presence or absence of structural defects [5]. A review of the literature reveals that in 20–28% of NIHF, cardiac anomalies are identified, followed by chromosomal abnormalities (13.5%), hematologic disorders (10.4%), and infections (6.7%); whereas in about 18–22% of the cases, despite thorough diagnostic evaluation including fetal chromosomal microarray and/or whole exome sequencing analysis and postnatal investigations, the cause remains unknown [4, 6–11].

The presence of hydrops fetalis in fetuses with cardiac anomalies, like structural heart defects, rhythm disturbances, cardiac tumors, and cardiomyopathies, is associated with poor prognosis. The perinatal mortality of these fetuses is high and has been reported up to 74% in the literature [12].

We decided to focus our interest on this subgroup of fetuses, because, in our own experience, the management of the pregnancy and the counseling of the family regarding prognosis is particularly challenging. Obviously, the severity of the underlying pathology as well as the presence of an underlying genetic abnormality are prognostic indicators of outcome [12, 13]. However, we have observed, that even though a certain cardiac anomaly leads to hydrops development in some cases, in others, it can be well tolerated by the fetus. We think that as long as myocardial function is sufficient, an expectant management attempting to prolong the pregnancy and reduce the risks of prematurity is beneficial for the fetus. Therefore, the key issue in the surveillance of these fetuses is to identify the right time to intervene with in utero fetal therapy, when the cause is treatable, or prompt delivery before fetal deterioration and demise occur. To date, however, there is no good prognostic indicator of fetal outcome and no ultrasonographic tool to help the physicians determine the appropriate timing of intervention.

Previous studies have used different ultrasonographic measurements and observations to evaluate fetal myocardial function and wellbeing in an attempt to predict fetal outcome [14–16]. Among them, the cardiovascular profile score (CVPS), proposed by Falkensammer et al., seems to be of value in the prediction of fetal congestive heart failure and therefore in the prediction of fetal outcome [14]. We used the CVPS as a tool to assess fetal myocardial function and aimed to explore its association with fetal outcome in this selected population of patients.

## Materials and Methods

In this retrospective study, we included fetuses with NIHF and a prenatally suspected cardiac anomaly based on ultrasound examination. The patients’ history and ultrasound data were reviewed.

A detailed ultrasound examination of fetal anatomy, including fetal echocardiography, was performed at presentation using a Voluson E8 Expert, (GE Medical systems, Solingen, Germany), a Voluson E10 Expert, (GE Medical systems, Solingen, Germany), or a Philips Epiq 5 ultrasound system (Hamburg, Germany), equipped with a 3.5 MHz and a 5 MHz transabdominal transducer. All data were saved in the ViewPoint ultrasound reporting and image archiving system (Solingen, Germany), which enabled retrospective analysis. M-Mode was used to assess fetal cardiac rhythm disturbances. Doppler interrogation was used for the evaluation of atrioventricular valve competence. Color flow Doppler was applied on the umbilical artery, umbilical vein, and ductus venosus. The pulsatility index of each vessel was calculated and compared to the reference values for normal fetuses of the same gestational age [17].

**Fig. 1:**
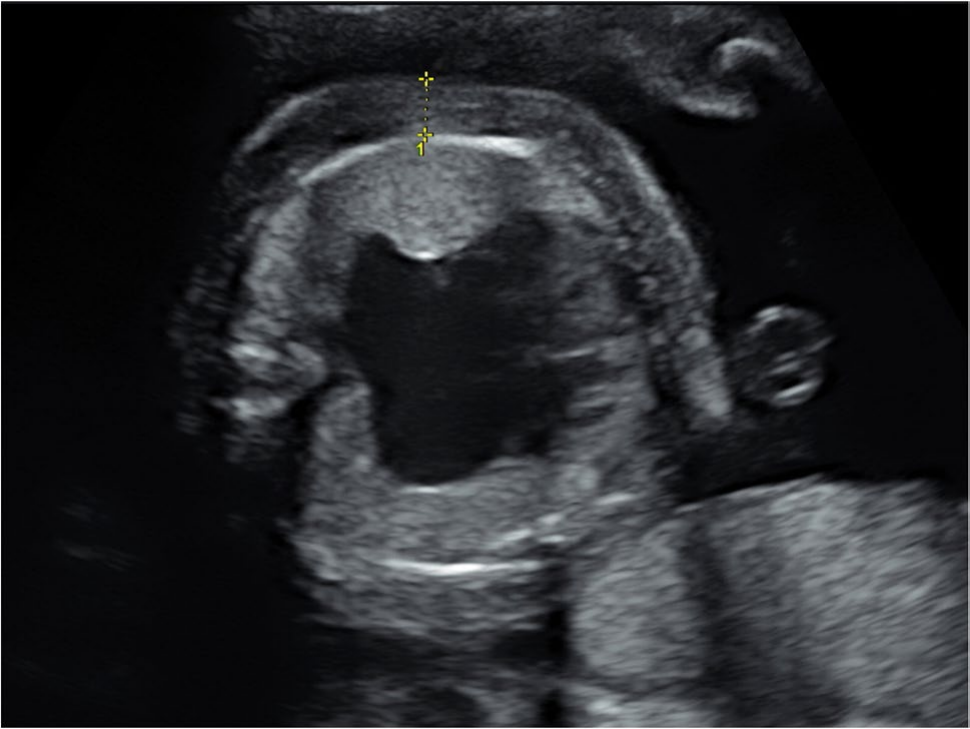
Case 1 at 24 + 1 weeks of gestation, skin edema measuring 7.4 mm, CVPS 5 points (skin edema: 0 points, mild cardiomegaly (cardiothoracic area ratio = 0.45): 1 point, mitral valve regurgitation: 0 points, normal arterial Doppler flow velocimetry: 2 points, normal venous Doppler flow velocimetry: 2 points)

**Fig. 2:**
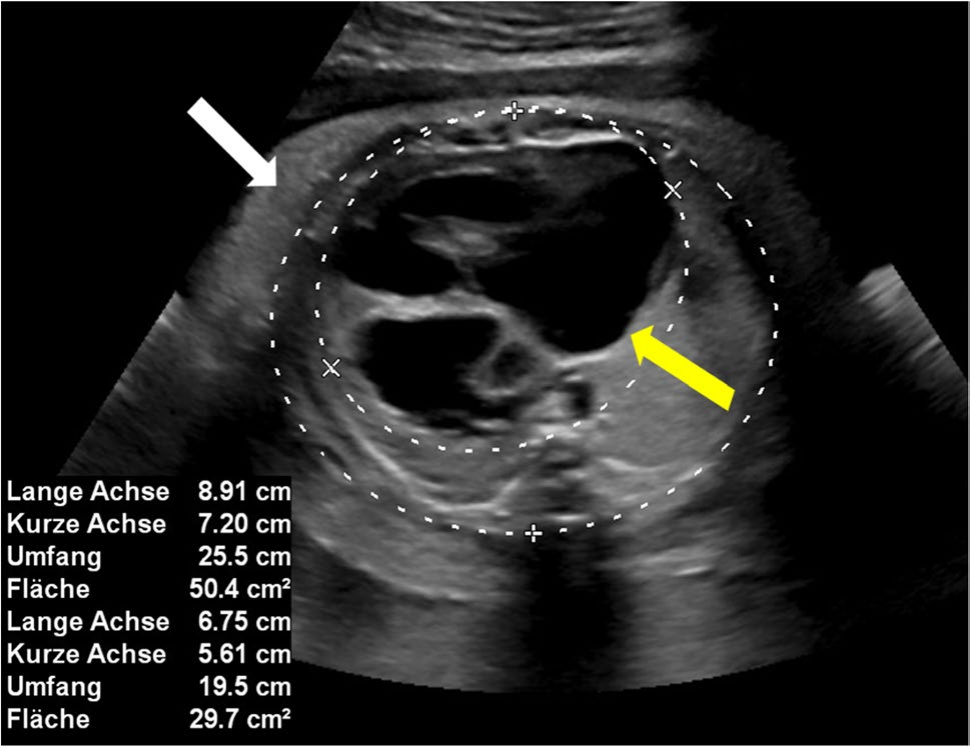
Case 3 at 23 + 4 weeks of gestation with severe cardiomegaly, CVPS 4 points, (ascites, pleural effusions: 1 point, severe cardiomegaly (cardiothoracic area ratio = 0.59): 0 points, tricuspid valve regurgitation: 1 point, normal arterial Doppler flow velocimetry: 2 points, pulsatile umbilical vein: 0 points), yellow arrow: heart, white arrow: skin edema

**Fig. 3:**
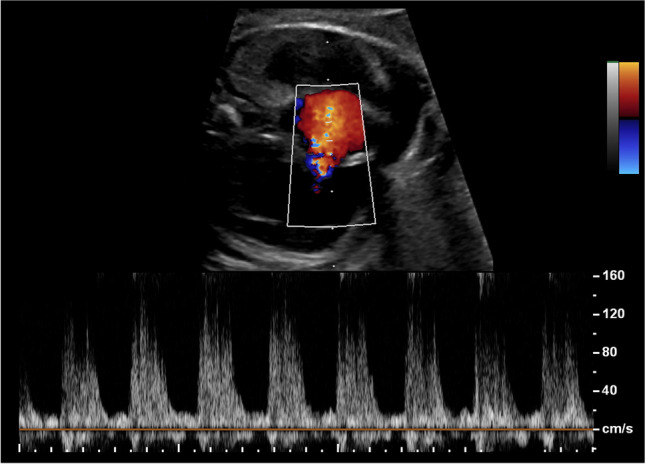
Case 3 at 23 + 4 weeks of gestation, here illustration of the holosystolic tricuspid valve regurgitation

**Fig. 4:**
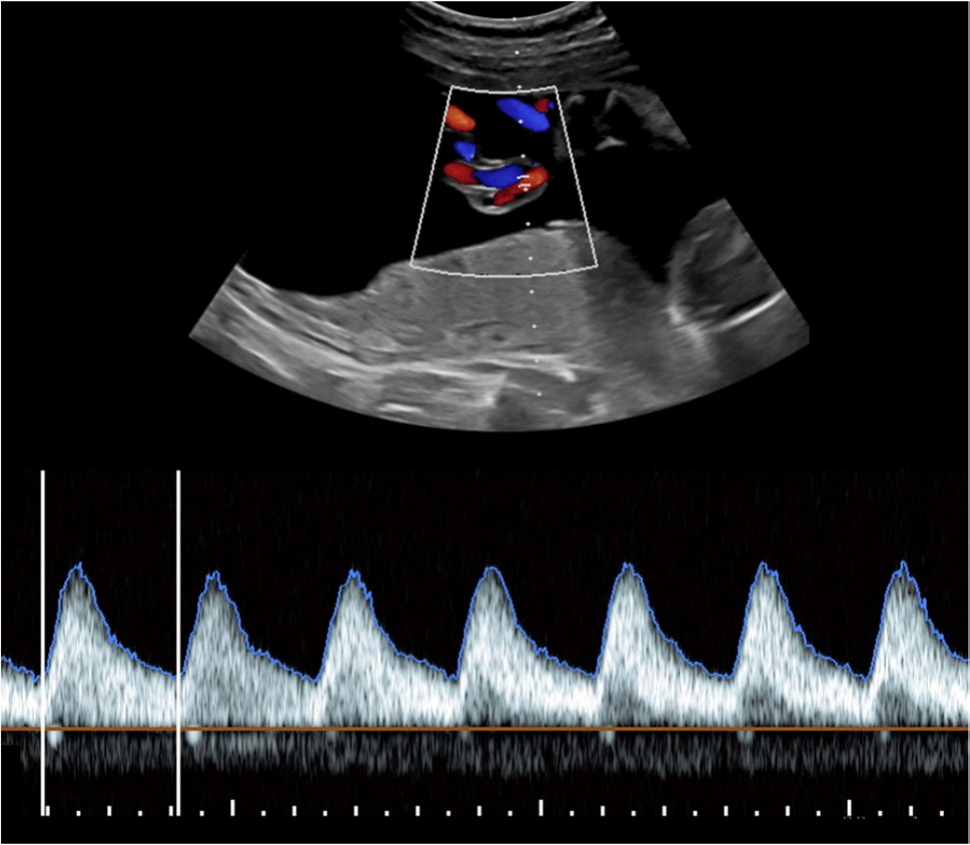
Case 11 at 30 + 0 weeks of gestation, CVPS 8 points (skin edema: 0 points, cardiothoracic area ratio = 0.28: 2 points, competent tricuspid and mitral valve: 2 points, normal arterial Doppler flow velocimetry: 2 points, normal venous Doppler flow velocimetry: 2 points)

**Fig. 5:**
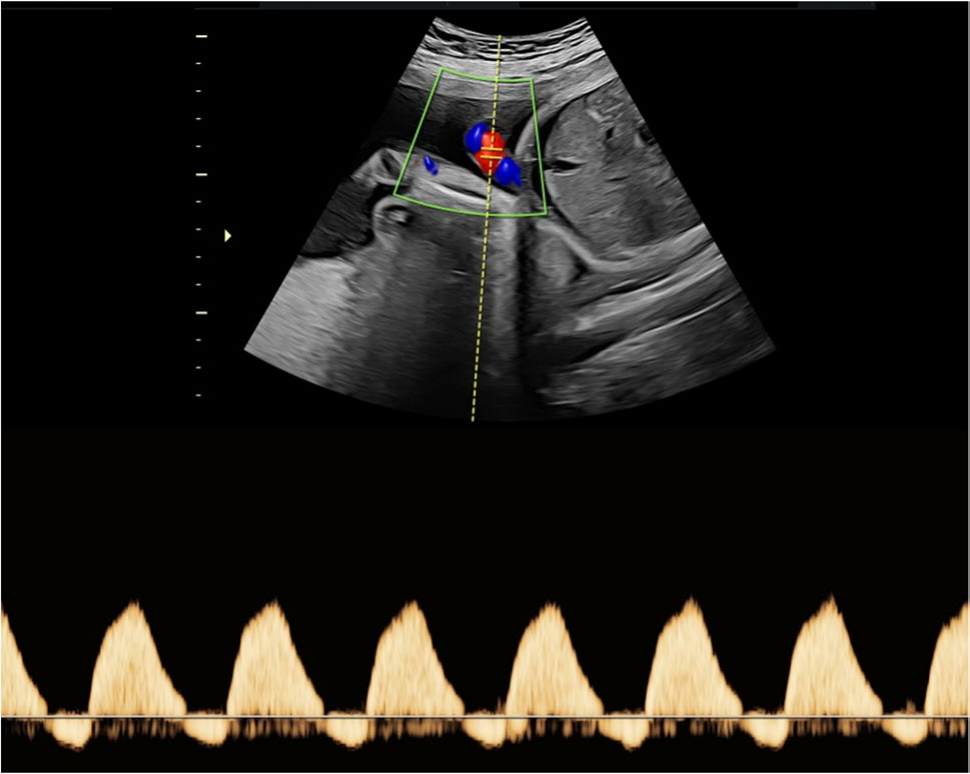
Case 2 at 31 + 6 weeks of gestation, CVPS 4 points (ascites, pleural effusions, generalized skin edema,: 0 points, mild cardiomegaly (cardiothoracic area ratio = 0.45): 1 point, competent tricuspid and mitral valve: 2 points, umbilical artery Doppler velocimetry with reverse end-diastolic flow: 0 points, ductus venosus with reversed a wave: 1 point)

**Fig. 6:**
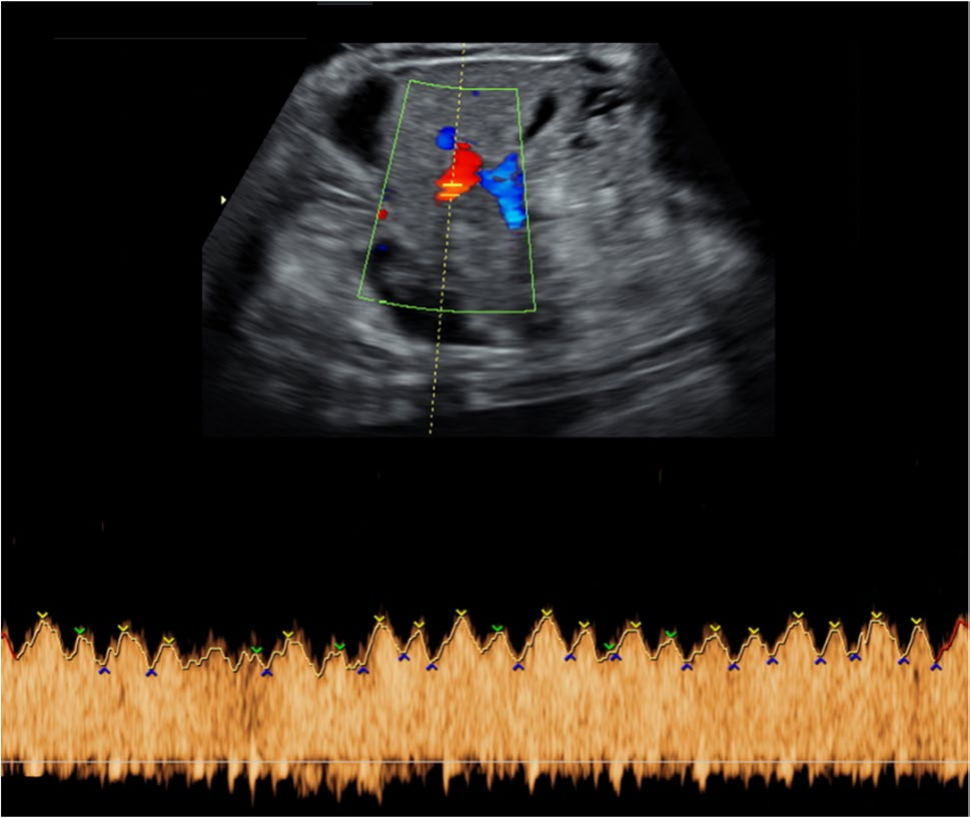
Case 9 at 32 + 6 weeks of gestation, CVPS 6 points (ascites, pleural effusions, generalized skin edema: 0 points, cardiothoracic area ratio = 0.2: 2 points, tricuspid valve regurgitation: 1 point, umbilical artery Doppler velocimetry with zero end-diastolic flow: 1 point, normal venous Doppler flow velocimetry: 2 points), yellow arrow: pleural effusion, white arrow: skin edema

**Fig. 7:**
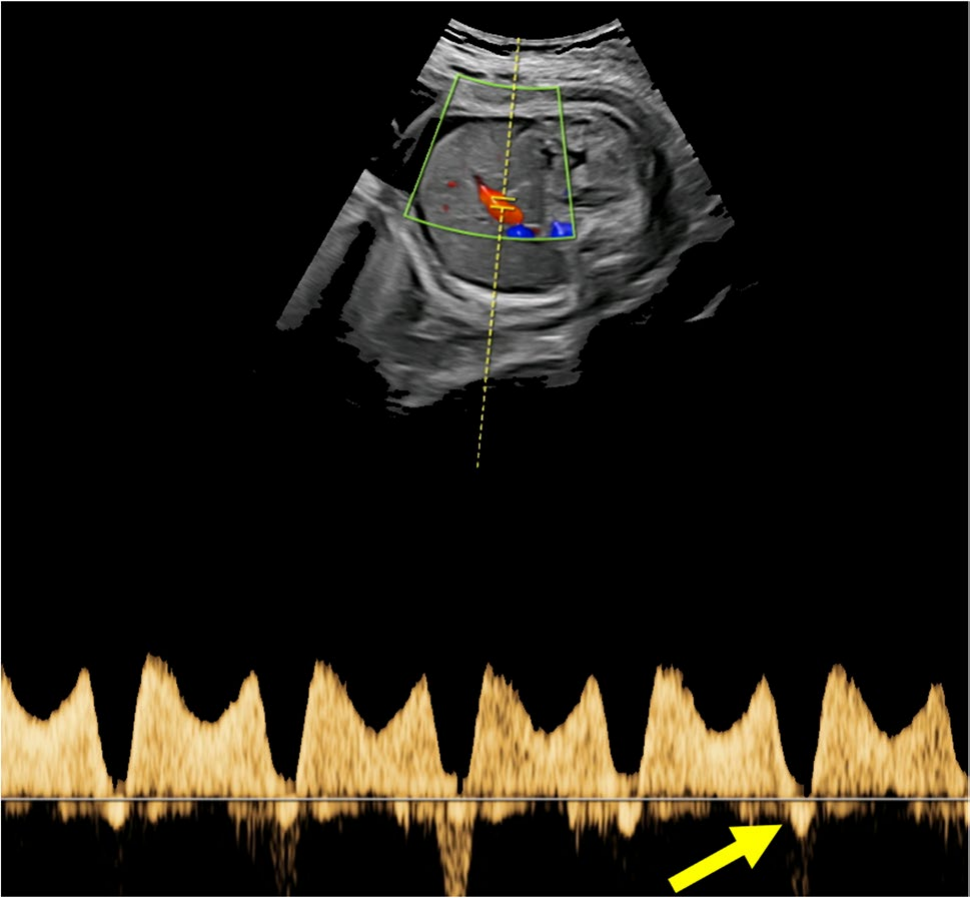
Case 2 at 31 + 6 weeks of gestation, here illustration of the blood flow pattern in the ductus venosus with reversed a wave (yellow arrow)

The CVPS was calculated using the information obtained by fetal echocardiographic examination. Five parameters were evaluated: (1) fetal hydrops, (2) cardiothoracic ratio, (3) pulsed Doppler study of the atrioventricular valves, (4) Doppler flow velocimetry of the umbilical artery, and (5) Doppler flow velocimetry of the ductus venosus and umbilical vein. Two points were attributed to each category for normal findings, meaning that the CVPS could achieve a maximum of ten points in a non-hydropic fetus with normal myocardial function. In case an abnormality occurred, the CVPS decreased by one or two points per category, depending on the severity of the findings as follows:

1. Fetal hydrops*:* One point was deducted for excessive fluid accumulation in the abdominal cavity (ascites), the pleura and/or the pericardium and two points for generalized skin edema (Fig. 1).
2. Cardiomegaly: The heart area was compared to the area of the thorax using the cardiothoracic area ratio (CTR, cardiac area/chest area, normal range 0.2–0.35) or the cardiothoracic circumference ratio (cardiac circumference/chest circumference, normal value < 0.5). One point was taken off for cardiothoracic area ratio 0.35–0.5 and two points for cardiothoracic area ratio or cardiothoracic circumference ratio > 0.5 (Fig. 2) [18].
3. Cardiac function: The cardiac function was assessed at the level of the atrioventricular valves. Two points were attributed to the fetus for a competent tricuspid and mitral valve. The presence of a non-holosystolic tricuspid valve regurgitation was also considered a normal finding. If holosystolic tricuspid regurgitation was documented, the score decreased by one (Fig. 3). Two points were taken off in cases with severe myocardial dysfunction with mitral valve regurgitation or monophasic ventricular diastolic filling.
4. Doppler velocity of the umbilical artery: The pulsatility index in the umbilical artery was calculated as a marker of redistribution of cardiac output. Two points were attributed to the score for a normal biphasic blood flow pattern in the umbilical artery with a positive end-diastolic flow (Fig. 4). One point was deducted from the score for an absent end-diastolic flow and two points for a reverse end-diastolic flow (Fig. 5).
5. Venous Doppler: The blood flow pattern in the ductus venosus and umbilical vein were assessed and the pulsatility index for veins (PIV) was calculated. Two points were attributed to the fetus for a normal triphasic pulsating pattern of the ductus venosus (Fig. 6) and a continuous non-pulsatile flow in the umbilical vein. The score decreased by one in case of an increase in the pulsatility index of the ductus venosus with zero or reverse flow during atrial contraction (a-wave) (Fig. 7) and by two if, additionally, umbilical vein pulsations were documented.

Parental counseling regarding diagnosis, anticipated prognosis and recommendations on further fetal surveillance was done by an interdisciplinary team, consisting of a specialist in perinatal medicine, a neonatologist and a pediatric cardiologist.

Primary outcome of the study was the feto-neonatal mortality (FNM), defined as intrauterine fetal demise (excluding termination of pregnancy) or death in the first 6 months of life. The CVPS was calculated once per fetus.

### Statistical Analysis

Gestational age in the population is described by its median. A permutation test was performed to test the null hypothesis that the distribution of CVPS does not differ between surviving fetus and fetus that died at a two-sided significance level of 5%.

## Results

Between January 2007 and March 2021, 98 patients with the diagnosis of NIHF were referred to the Department of Obstetrics and Gynecology of the University Medical Center of the Johannes Gutenberg University Mainz, Germany. By eighteen of them, the suspicion of a cardiac anomaly in prenatal ultrasound was raised. After exclusion of six pregnancies (one termination of pregnancy and five because of incomplete data), twelve cases were left for analysis. The median maternal age was 31 years. Mean gestational age at which the CVPS was calculated was 29 + 2 weeks. Two fetuses died in utero (case 1, CVPS 5, and case 2, CVPS 4). Of the remaining ten hydropic fetuses, three newborns died in the neonatal period, and seven survived after a 6 months surveillance period. Median CVPS of all fetuses was 6. Surviving fetuses (*n* = 7) showed statistically significantly higher CVPS values (median 8) than fetuses who died (*n* = 5) (median 5, *p* value = 0.009).

All liveborn fetuses were admitted to the neonatal unit of our department and received an echocardiographic examination, which in all but one case confirmed the prenatal diagnosis.

The perinatal course, CVPS values and postnatal diagnosis of each fetus are summarized in Table 1.

**Table 1:** Information on perinatal course

	GA at which the CVPS was calculated	GA at delivery	Apgar score (1, 5, 10 min)	Diagnosis	CVPS	Outcome 1 = survived 0 = FNM
1	24 + 1w	26 + 0w	0/0/0	AV atresia, restrictive FO	5	0
2	31 + 6w	34 + 4w	0/0/0	Common arterial trunk	4	0
3	23 + 4w	36 + 4w	4/3/4	LVNC	4	0
4	30 + 2w	32 + 2w	3/6/7	LVNC	6	0
5	26 + 4w	30 + 2w	5/7/8	DORV with pulmonary stenosis, 22q11.2 deletion syndrome	5	0
6	28 + 5w	31 + 0w	4/7/9	PSVT	9	1
7	33 + 5w	36 + 1w	7/5/8	Pseudo-atresia of the PV, TV dysplasia	6	1
8	25 + 0w	39 + 5w	9/10/10	CMV myocarditis, dilatative cardiomyopathy	9	1
9	32 + 6w	32 + 6w	2/3/6	Left pulmonary artery stenosis, Trisomy 21	6	1
10	31 + 5w	35 + 5w	4/7/8	PSVT	7	1
11	30 + 0w	30 + 1w	1/3/4	DORV, Noonan syndrome	8	1
12	32 + 1w	32 + 2w	5/6/8	Suspicion of heart defect was not confirmed postnatally	9	1

*GA* Gestational age, *CVPS* cardiovascular profile score, *FNM* feto-neonatal mortality, *w* weeks of gestation, *AV* aortic valve, *FO* foramen ovale, *LVNC* left ventricular non-compaction cardiomyopathy, *DORV* double outlet right ventricle, *PSVT* paroxysmal supraventricular tachycardia, *PV* pulmonary valve, *TV* tricuspid valve, *CMV* cytomegalovirus

Table 2 illustrates the sonographic findings and the CVPS values for each case respectively.

**Table 2:** Sonographic findings and CVPS values

Case	NIHF 1:ascites, pleura-/pericard-effusion 0:skin edema	Cardiomegaly 2:CTR ≤ 0.35 1:CTR 0.35–0.5 0:CTR > 0.5	Cardiac function 2:n 1:TR 0:MR or monophasic ventricular diastolic filling	UA PI 2:n 1:AED 0:RED	Venous Doppler 2:n 1:increased DV PIV 0:UV pulsations	CVPS
1	0	1	0	2	2	5
2	0	1	2	0	1	4
3	1	0	1	2	0	4
4	0	1	2	2	1	6
5	1	1	0	2	1	5
6	1	2	2	2	2	9
7	1	0	1	2	2	6
8	1	2	2	2	2	9
9	0	2	1	1	2	6
10	0	2	1	2	2	7
11	0	2	2	2	2	8
12	1	2	2	2	2	9

*NIHF* Non-immune hydrops fetalis, *CTR* cardiothoracic area ratio, *n* normal, *TR* tricuspid valve regurgitation, *MR* mitral valve regurgitation, *UA* umbilical artery, *PI* pulsatility index, *AED* absent end-diastolic flow, *RED* reverse end-diastolic flow, *DV* Ductus venosus, *PIV* pulsatility index for veins, *UV* umbilical vein, *CVPS* cardiovascular profile score

With regard to perinatal outcome, one fetus with aortic valve atresia (case 1) and one fetus with common arterial trunk died in utero (case 2). Among the remaining three fetuses with FNM who were born alive, two were diagnosed with left ventricular non-compaction cardiomyopathy and died at the age of 6 weeks (case 3) and 4 months (case 4), respectively. The third fetus had 22q11.2 deletion syndrome with double outlet right ventricle (DORV) and pulmonary stenosis and died at two months of age (case 5).

Six fetuses (three survivors (cases 9–11) and three non-survivors (cases 1, 2, und 4)) developed severe hydrops with skin edema. Cardiomegaly occurred in all fetuses with FNM (cases 1–5). On the contrary, only one of the surviving fetuses who was diagnosed with pseudo-atresia of the pulmonary valve and tricuspid valve dysplasia, developed severe cardiomegaly in utero (case 7). The child survived after surgical treatment. Normal cardiac function was found in four out of seven fetuses who survived (cases 6, 8, 11, and 12) and in two cases with FNM (cases 2 and 4). Zero flow in the umbilical artery occurred in one fetus with left pulmonary artery stenosis and Trisomy 21 that survived (case 9). Reverse end-diastolic flow in the umbilical artery occurred in one case. The fetus was diagnosed with common arterial trunk, developed severe hydrops and died in utero (case 2). Doppler velocimetry study of the Ductus venosus showed a normal triphasic pattern in all survivors. Increased Doppler flow velocimetry of the Ductus venosus was identified in four out of five non-survivors (cases 2, 3, 4, and 5), one of whom also showed umbilical venous pulsations (case 3).

## Discussion

In this pilot study, we could point towards a positive correlation between the cardiovascular profile score and fetal outcome in a selected population of patients with non-immune hydrops fetalis (NIHF) and cardiac anomalies. We believe that as long as myocardial function is sufficient, an expectant management in order to prolong the pregnancy and reduce the risks of prematurity is beneficial for the fetus.

Structural heart defects, rhythm disturbances, cardiac tumors, cardiomyopathies, and myocarditis are all anomalies that can lead to the development of hydrops fetalis [19]. The pathogenesis of NIHF with cardiac anomalies is complex and not completely understood. Previous studies provide data that in this subgroup of fetuses, the primary cause of hydrops development is myocardial dysfunction and cardiac decompensation. It has also been shown that myocardial dysfunction may precede the clinical state of hydrops [14, 18]. The final pathway through which cardiac anomalies lead to NIHF and eventually fetal demise is considered to be inadequate tissue perfusion because of decrease of cardiac output and subsequently development of congestive heart disease (CHD) [15]. Once cardiac decompensation occurs, increased ventricular end-diastolic pressure subsequently leads to an elevation of central venous pressure. The fetal lymphatic flow is particularly susceptible to changes in the outflow pressure. As a result, even a small increase in central venous pressure results in a remarkable decrease in lymphatic return. Decreased lymphatic return, in combination with an increased permeability of fetal capillaries, lead to an excessive fluid movement to the interstitial compartment, and eventually to hydrops [7].

After the etiology of NIHF is established or suspected, the most challenging part is the management of the pregnancy. The determination of the right time to intervene with delivery or fetal therapy before fetal demise occurs is of utmost importance during the surveillance of these fetuses. However, clinical guidelines indicating how to manage the pregnancy and how to determine the delivery timing are lacking in the literature.

In order to evaluate myocardial function and predict fetal outcome, the assessment of different parameters and indexes has been proposed. For example, measurements of ventricular shortening fraction have been used to evaluate cardiac contractility. Togsong et al. used cardiospatiotemporal imaging with M-mode technology in order to create reference ranges for ventricular shortening fraction in normal fetuses. When the measurements were performed in fetuses with NIHF and congenital heart defects, a significant decrease in both right and left ventricular shortening fraction could be demonstrated. The authors concluded that the cardiac pathology leads to impaired cardiac contractility, development of congestive heart disease, and eventually to hydrops fetalis [15].

Eidem et al. defined normal values for the myocardial performance index (MPI) or Tei-Index in normal fetuses between 20 and 40 weeks of gestation. The Tei-Index, which is defined as the ratio of the isovolemic time to the ejection time of a ventricle, is a non-invasive echocardiographic method for assessing global ventricular performance in adult and pediatric population [20]. In fetuses with hydrops fetalis, the Tei-Index of both, the right and left ventricle was found to be significantly increased, indicating fetal congestive heart failure [14].

Compared to the abovementioned approaches, the CVPS has the advantage of combining five different parameters that are all associated with fetal wellbeing, and are easy to be obtained even by a non-specialist in fetal echocardiography. Particularly in settings where the expertise in fetal echocardiography is lacking, the CVPS can help estimate the severity of the disease, before further decisions are made (for example immediate delivery or transfer of care in a tertiary center).

The CVPS was initially introduced by Falkensammer et al. Further, Hofstaetter et al. used it to evaluate myocardial function in 102 fetuses with NIHF of various etiologies [21]. In their study, fetal outcome was defined as prenatal death or death within the first 7 days of life. Twenty-one pregnancies were terminated. From the remaining 81 fetuses, 54 (67%) survived, and 27 (33%) died perinatally. Similar to our findings, fetuses who died prenatally or postnatally had lower CVPS values (median 6), than survivors (median 7). Venous Doppler sonography was found to be the best predictor of adverse outcome. The authors concluded that the CVPS can be used in the surveillance of hydropic fetuses and as an aiding tool for predicting fetal outcome. The main difference between our study and the study of Hofstaetter et al. is that the latter included fetuses with NIHF of various etiologies, whereas we chose to specifically examine those NIHF cases with a prenatally suspected cardiac anomaly.

Few studies have applied the CVPS on fetuses with congenital heart disease (CHD). Miyoshi et al. used the CVPS in order to grade the severity of fetal congestive heart failure in 202 fetuses with CHD. Even though the study does not specify what percentage of the investigated fetuses manifested hydrops, it demonstrated that the CVPS is a useful marker in predicting fetal and neonatal outcome and that CVPS ≤ 5 is an independent predictor of perinatal mortality [22]. Similar results were demonstrated by Wieczorek et al. in a retrospective study of 131 singleton pregnancies diagnosed with CHD. Even though this study did not include fetuses with extremely low CVPS, a score ≥ 8 was associated with good perinatal outcome [23].

The surveillance of fetuses with NIHF and cardiac anomalies represents a challenge for both obstetricians and neonatologists. Guidelines and management protocols are lacking in the literature. In this retrospective study, we used the CVPS to grade the severity of fetal congestive heart failure in fetuses with NIHF and cardiac anomalies. Although our study was limited to a small number of patients, our results point towards a positive correlation between CVPS and fetal outcome in this selected population of fetuses.

The main limitations of this study are the small sample size, mainly due to the rarity of the disease, and its retrospective character. The ultrasound examinations were carried out as part of the clinical consultation and not for the purposes of the study. This resulted in having to exclude patients because of incomplete data. We acknowledge that, in some cases, the presence of an underlying pathology, like a genetic abnormality, in addition to the presence of a cardiac anomaly, will affect the development of hydrops fetalis and eventually fetal outcome. However, we believe that as long as myocardial function is sufficient, it is beneficial to attempt to prolong the pregnancy in order to reduce the risks of prematurity. What still remains unclear is, whether delivery improves the neonatal outcome in cases with very compromised fetuses and low CVPS values. Larger prospective studies are needed in order to address this issue.

In our unit, we have integrated the CVPS in the surveillance of all hydropic fetuses, and we are trying to further investigate its use as a prognostic marker. We are also trying to address the question in what way and with which cut off values the CVPS can be implemented in the surveillance of hydropic fetuses and be used an aiding tool to guide delivery decisions. Further, we are applying the CVPS on fetuses with cardiac anomalies without hydrops, since we believe that serial CVPS measurements can be of value on the surveillance of these fetuses as well.

In conclusion, in fetuses with NIHF and cardiac anomalies, the CVPS appears to correlate with fetal outcome and to be a useful marker in the assessment of heart failure in utero. Especially in settings with low expertise in fetal echocardiography, the CVPS can be used as a tool to help estimate the severity of fetal compromise before further decisions are made (e.g., if there is time to transfer the patient to a tertiary center). Furthermore, the findings of this study could be useful for counseling prospective parents.

## Data Availability

The relevant data have been included in the manuscript, the raw data are not provided because of concerns regarding patient anonymity. Access to the raw data can be provided upon reasonable request on the corresponding author.
